# Supplementary figures and images for: Soil microbial gene expression in an agricultural ecosystem varies with time and neonicotinoid seed treatments
Source: Microbiology (Reading). 2023 Apr 21;169(4):001318. doi: 10.1099/mic.0.001318 (PMC10202318; doi:10.1099/mic.0.001318)

Figure S1

A)

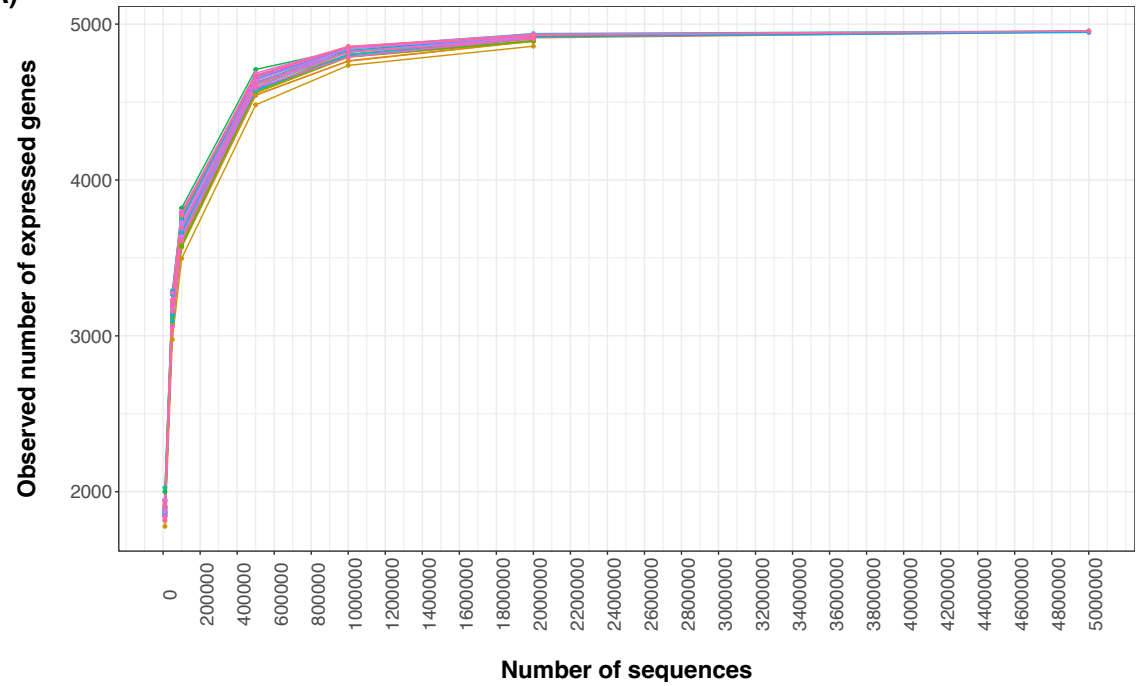

B)

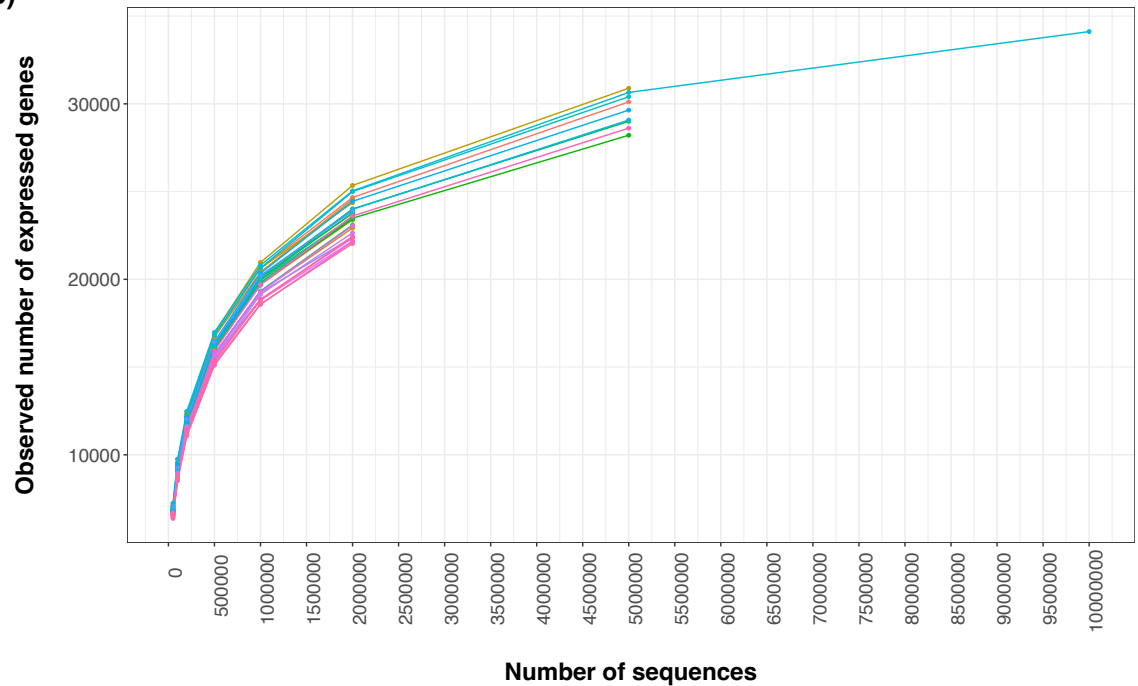

C)

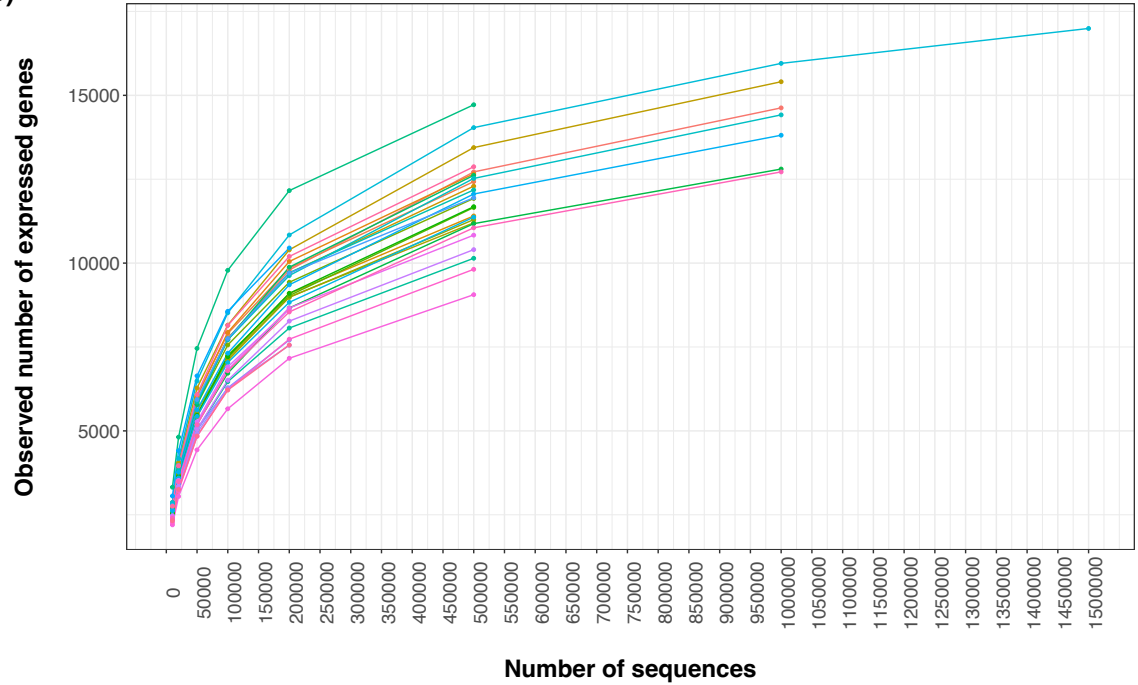

Figure S2

A)

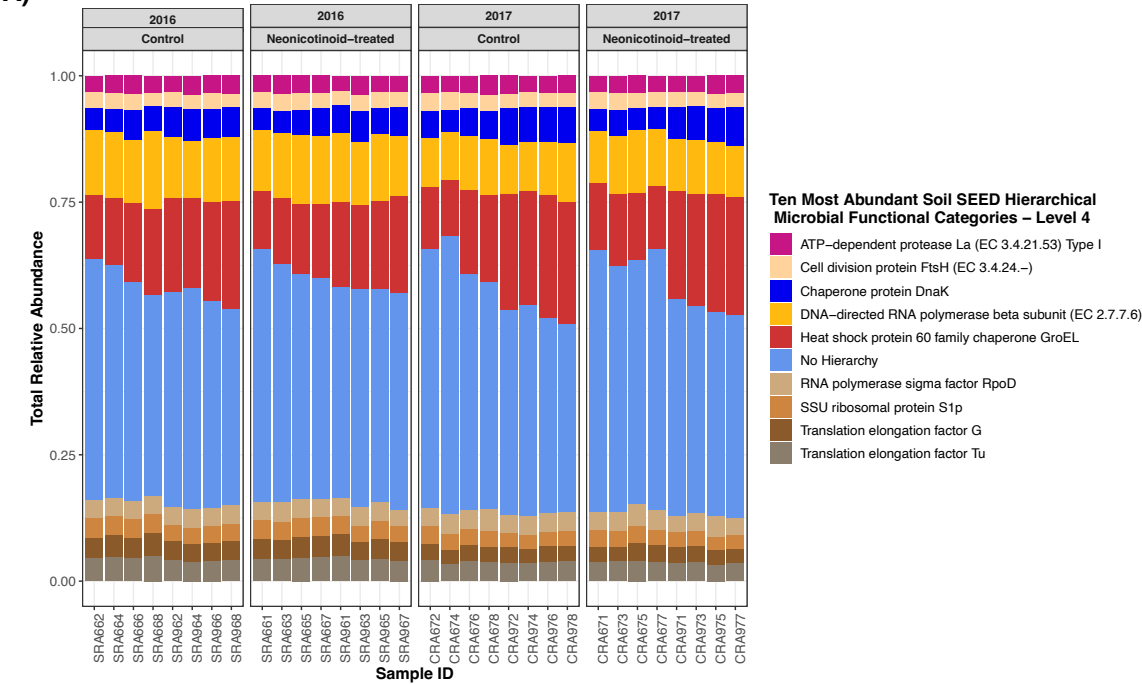

B)

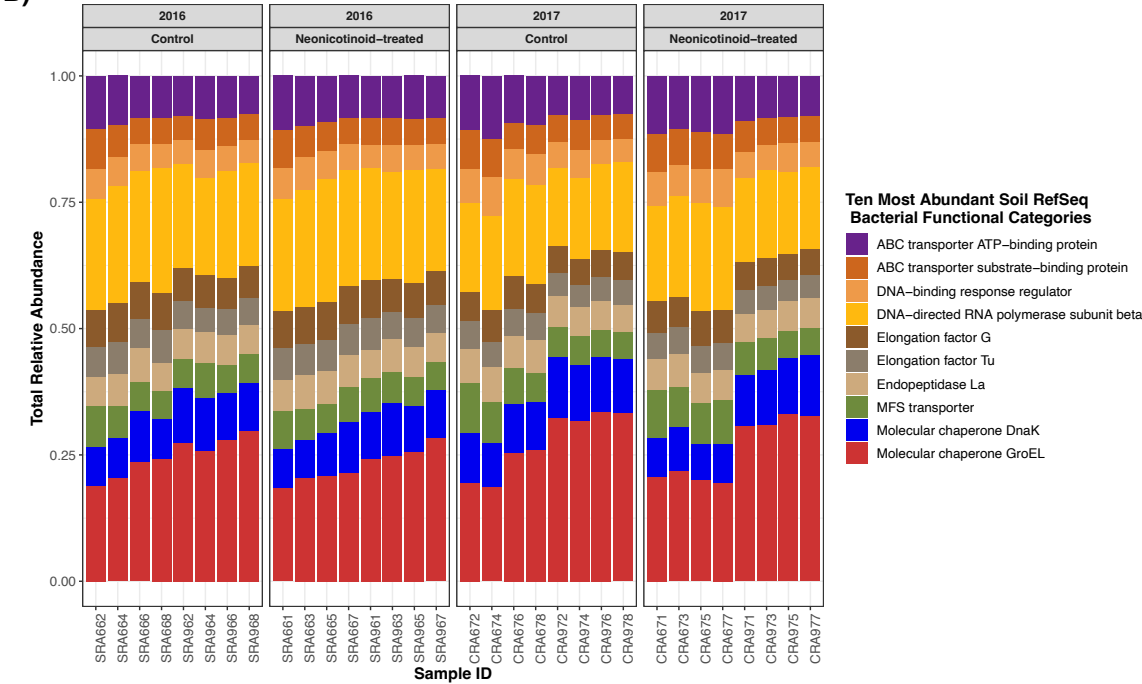

C)

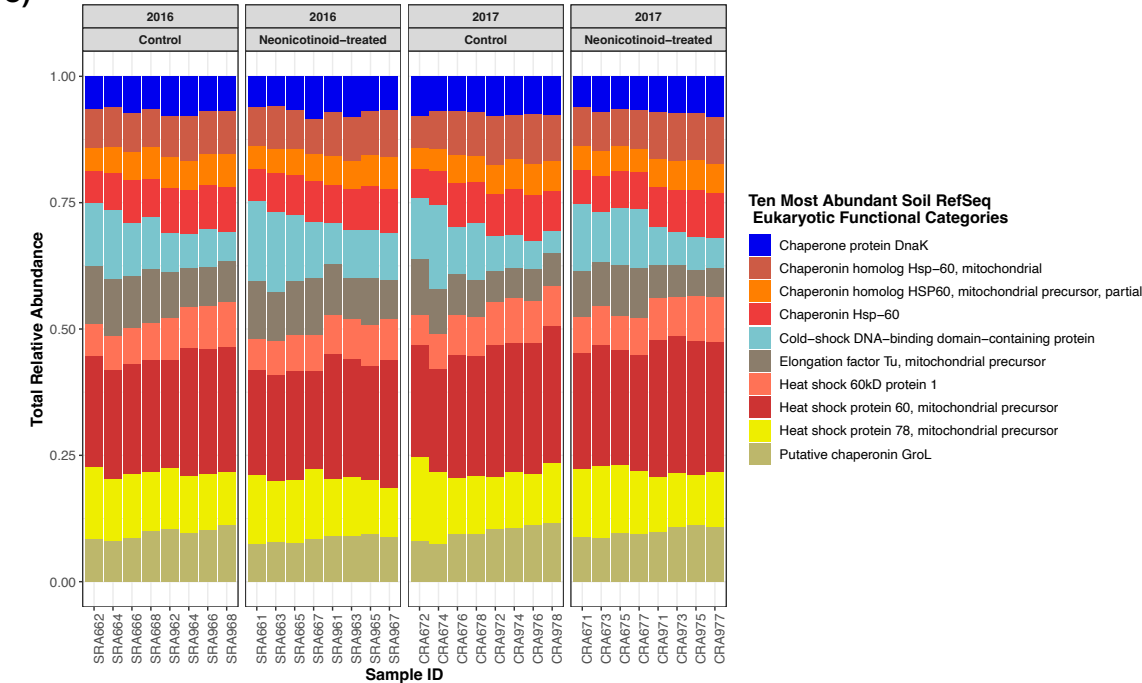

Supplement: Supplementary material 1 [file mic-169-1318-s001.pdf]
